# Residential Mercury Contamination in Adobe Brick Homes in Huancavelica, Peru

**DOI:** 10.1371/journal.pone.0075179

**Published:** 2013-09-10

**Authors:** Nicole Hagan, Nicholas Robins, Heileen Hsu-Kim, Susan Halabi, Ruben Dario Espinoza Gonzales, Daniel deB. Richter, John Vandenberg

**Affiliations:** 1 Department of Environmental Sciences and Engineering, the University of North Carolina at Chapel Hill, Chapel Hill, North Carolina, United States of America; 2 Department of History, North Carolina State University, Raleigh, North Carolina, United States of America; 3 Department of Civil and Environmental Engineering, Duke University, Durham, North Carolina, United States of America; 4 Department of Biostatistics and Bioinformatics, Duke University, Durham, North Carolina, United States of America; 5 Environmental Health Council, Huancavelica, Peru; 6 Nicholas School of the Environment, Duke University, Durham, North Carolina, United States of America; 7 Office of Research and Development, U.S. Environmental Protection Agency, Research Triangle Park, North Carolina, United States of America; Kagoshima University Graduate School of Medical and Dental Sciences, Japan

## Abstract

This is the first study of adobe brick contamination anywhere in the world. Huancavelica, Peru is the site of historic cinnabar refining and one of the most mercury (Hg) contaminated urban areas in the world. Over 80% of homes in Huancavelica are constructed with adobe bricks made from Hg contaminated soil. In this study we measured total Hg concentrations in adobe brick, dirt floor, surface dust, and air samples from the interior of 60 adobe brick houses located in four neighborhoods. Concentrations of total Hg in adobe bricks, dirt floors, and surface dust ranged from 8.00 to 1070 µg/g, 3.06 to 926 µg/g, and 0.02 to 9.69 µg/wipe, respectively, with statistically significant differences between the four neighborhoods. Concentrations of Hg in adobe brick and dirt floor samples in Huancavelica were orders of magnitude higher than in Ayacucho, a non-mining town in Peru. A strong correlation exists between total Hg concentrations in adobe bricks and dirt floors which confirms that adobe bricks were being made on-site and not purchased from an off-site source. A strong correlation between surface dust and adobe bricks and dirt floors indicates that walls and floors serve as indoor sources of Hg contamination. Elemental Hg vapor concentrations were below detection (<0.5 µg/m^3^) in most homes; however in homes with detectable levels, concentrations up to 5.1 µg/m^3^ were observed. No statistically significant differences in Hg vapor measurements were observed between neighborhoods. This study demonstrates that building materials used widely in developing communities, such as adobe bricks, may be a substantial source of residential Hg exposure in silver or gold refining communities where Hg is produced or used for amalgamation in artisanal gold production.

## Introduction

Legacy mercury (Hg) contamination resulting from cinnabar mining and refining, particularly in ambient soils, has been studied in various parts of the world, most notably Almadén, Spain [1,2] and Idrija, Slovenia [3]. In Almadén, Hg concentrations in ambient soil have varied across studies, ranging from less than 1 µg/g [2] to over 8800 µg/g [1], while in Idrija measured concentrations have ranged from less than 1 to over 2700 µg/g [3]. Huancavelica was the site of one of the largest urban cinnabar refining operations, yet legacy contamination was not studied in the city until 2009 [4,6,7]. In a previous study, we reported Hg concentrations in ambient soil ranging up to 1200 µg/g [4], among the highest concentrations noted in the literature. It is important to understand the extent of this contamination and characterize the Hg present to estimate potential risks to the local populations from inhalation of Hg offgassing as vapor or from inhalation or ingestion of contaminated soil particles. This issue is especially important for children who tend to ingest more soil and dust than adults as a result of hand to mouth activity.

Huancavelica, Peru is located at an elevation of 3660 m in the Andes, where for over 350 years it served as the primary source of Hg used for amalgamation-based silver production in South America [5,6]. The 1563 discovery of cinnabar ore (HgS) in the Santa Barbara Hill just outside of Huancavelica provided a vast economic resource for Spain because Hg was essential to silver refining. To refine Hg, extracted cinnabar ore was crushed and smelted, a process in which the Hg was volatilized, collected, and shipped to Andean silver mining centers [6]. Extensive environmental contamination occurred in Huancavelica because the Hg vapor and liquid would routinely escape from the inefficient smelters located in the city. Based on colonial records, 17,000 metric tons of Hg vapor were emitted in Huancavelica between 1564 and 1810 [6,7]. Most of the Hg vapor likely deposited in and around the city, resulting in extremely high levels of urban contamination.

In Huancavelica, exposure to Hg contamination may be intensified compared to other cinnabar refining areas like Almadén and Idrija. Huancavelica is the capital of the most impoverished department of Peru where its 42,000 inhabitants live with the toxic legacy of over 400 years of Hg contamination. Most residents are not aware of the high levels of Hg to which they may be exposed or the health effects of Hg contamination; those who are informed often lack the resources to alleviate the problem. Health effects may be exacerbated as more than 80% of homes in the city are constructed with adobe bricks fabricated on-site from contaminated soil [8]. Problems arise because the interior walls and floors are generally unsealed, allowing Hg to emanate in both particulate and vapor form into what is usually a poorly ventilated space. Unlike residents of Almadén and Idrija, who live in homes constructed using modern materials such as masonry brick, residents in Huancavelica are exposed to Hg not only from ambient contamination but also from the contaminated interior walls and floors of their homes. This study was undertaken to determine the levels of Hg to which the current residents of adobe brick homes in Huancavelica are exposed. Its goal was to better understand the primary sources of Hg within residences, and the interrelationships among these sources.

The results of this study have broad application to other areas in the world that use Hg for artisanal silver and gold refining and that construct buildings and homes from adobe brick, such as Madre de Dios and Puerto Maldonado, both also in Peru. It is important to identify the primary indoor sources of Hg and the distribution of contamination across the city with respect to historic sources, both to characterize exposure and potential health outcomes, as well as to develop and evaluate mitigation and remediation efforts.

## Methods

**Figure 1:**
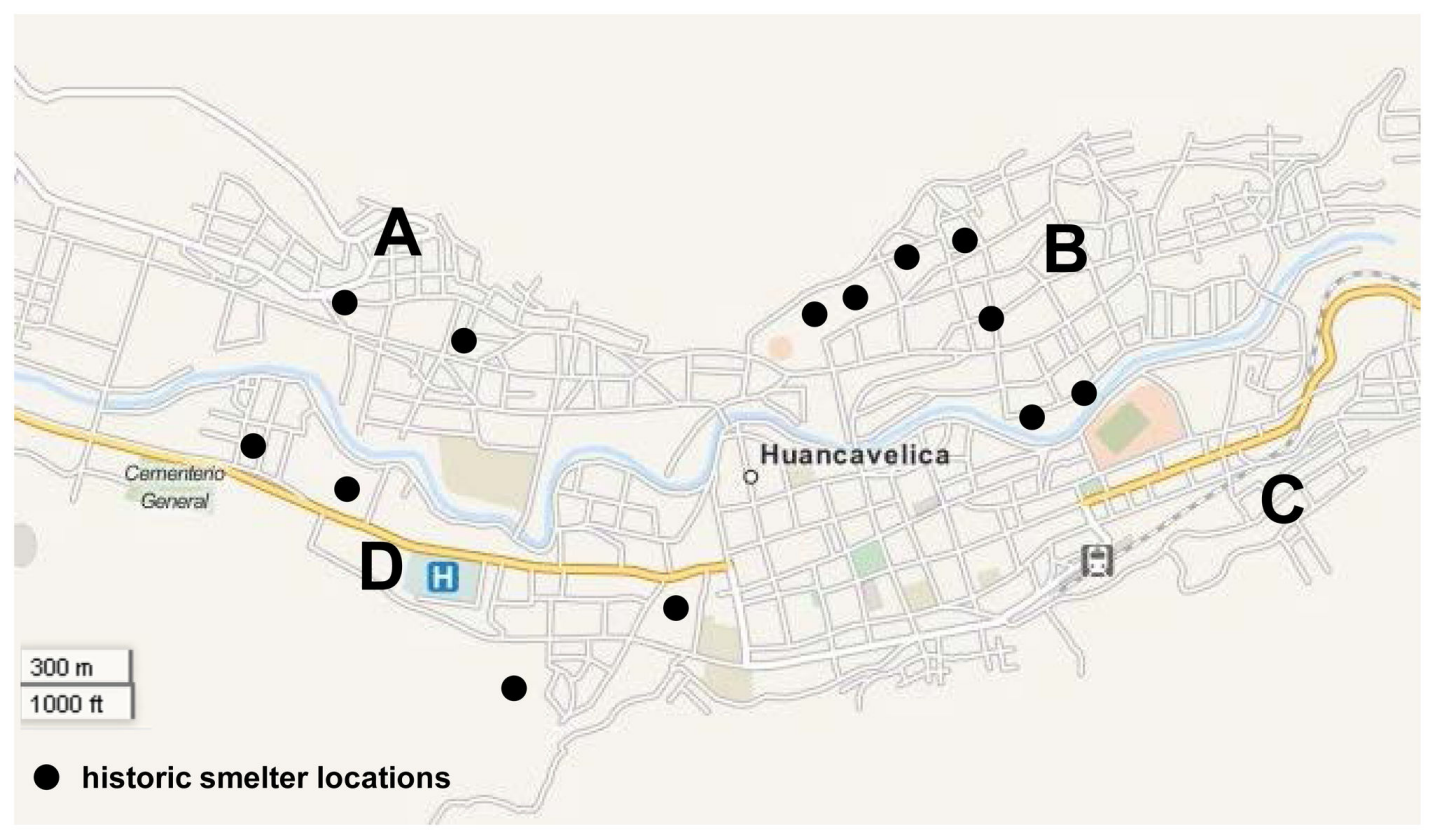
Map of historic smelter locations and neighborhoods sampled in 2010 in Huancavelica, Peru.

Figure 1 shows a map of the historic smelter locations and neighborhoods in Huancavelica. We expected to find higher Hg concentrations in samples from homes located in neighborhoods with historic cinnabar smelters (Ascencion (A), San Cristóbal (B), and Yananaco (D), shown in Figure 1) as compared to Santa Ana (C), a neighborhood with no historic cinnabar refining. We also expected to find similar concentrations of Hg in adobe bricks and dirt floors, assuming adobe bricks were made on-site. Moreover, we expected Hg concentrations in surface dust to be correlated with concentrations in adobe bricks and dirt floors, as we hypothesized that surface dust was primarily coming from indoor sources. We did not expect to find significant differences in Hg vapor concentrations between neighborhoods because vapor is relatively mobile.

### Sample collection

Samples of interior adobe brick, dirt floor, surface dust, and vapor were collected from 60 residences in Huancavelica, Peru in August 2010. These samples were collected from 15 homes in each of the four neighborhoods, Ascención, San Cristóbal, Santa Ana and Yananaco, shown in Figure 1. These neighborhoods were selected based on the historical location of cinnabar smelters and the results of previous ambient soil sampling [4]. Participants were recruited from public community meetings and interactions; verbal consent for participation was given by an adult resident or homeowner. Consent was recorded through the inclusion of the participant on the field sampling log. Institutional review board (IRB) approval for the study and verbal consent was obtained through Duke University.

In each home, samples were collected from the room where residents spent the most time. Triplicate samples were collected from the same room of each house. Adobe brick samples were obtained by scraping approximately 20 g of surface material to a depth of 2.5 cm from the interior adobe brick walls from three locations in the room. Dirt floor samples were collected by removing approximately 20 g of surface soil from the floor to a depth of 2.5 cm from three locations in the same room. In homes that had solid sealants on floors (e.g., wood or concrete), floor samples were obtained from the dirt patio immediately outside of the home (less than 10% of homes in the study). Triplicate samples of the adobe brick walls and dirt floors were not pooled; each sample was stored individually in a specimen bag. Surface dust samples were obtained by wiping three separate 100 cm^2^ areas of a hard, flat surface (e.g., kitchen table, dresser) with three moistened smear tabs (Whatman Low Ash Grade 50 Filter Paper, Fisher Scientific). Each smear tab was folded with the sample side inward and stored in a separate specimen bag. Once transported, all samples were stored at 4 °C in the laboratory until analysis.

Control samples were collected in Ayacucho, a non-mining town in Peru, located about 150 km southeast of Huancavelica. Triplicate samples of exterior adobe brick scrapings were collected from three homes and duplicate samples of ambient soil were collected from five homes using the same sampling and storage methods used in Huancavelica.

In situ mercury vapor measurements were recorded using a Jerome J405 Mercury Vapor Analyzer (Arizona Instrument LLC, Chandler, AZ). The instrument was purged outside each home prior to collecting measurements indoors. Three measurements were recorded for each household. Measurements below the limit of detection (LOD, 0.5 µg/m^3^) were set to LOD divided by the square root of 2 (resulting in 0.35 µg/m^3^ used for below LOD samples) when averaging the data.

### Sample preparation and Hg analysis

Adobe brick, dirt floor, and surface dust wipe samples were digested by hot block extraction in 4:1 HCl:HNO_3_ at 85°C for five hours. After digestion, the extract was diluted in 1% bromine monochloride and analyzed for total Hg content by stannous chloride reduction, gold amalgamation, cold vapor atomic fluorescence spectrometry (EPA Method 1631) [9] using a Brooks Rand MERX-T (Brooks Ran Labs, LLC, Seattle, WA). Instrument calibrations were performed with an acidified mercuric nitrate stock solution. A NIST-certified standard reference material (2709 San Joaquin soil) was digested in parallel with each batch of samples. The average recovery of total Hg in the standard reference material (SRM) was 107 ± 14% (n=21). Recovery of Hg from the SRM was within the acceptable range of the certified value (1.4 ± 0.08 µg/g).

### Statistical analysis

Total Hg concentrations were tested for normality and were found to be non-normally distributed; therefore, the data were log-transformed prior to statistical analyses. Diagnostic tests for fit (scatter plots of residuals, absolute residuals, and observed responses by predicted values; studentized residuals by leverage; Cook’s D by observation; a Q–Q plot of residuals; a residual histogram; and a residual-fit spread plot) indicated that there were no true outliers in the data set following log-transformation. Results of a three-way analysis of variance (ANOVA) suggested that differences in Hg concentrations for all sample types were driven by neighborhood and household variability. Statistically significant differences were not found between the triplicate samples; therefore, concentrations of Hg in all triplicate samples were averaged for each sample type within a home.

Results shown in figures and tables are arithmetic means. Descriptive statistics were computed and boxplots were used to summarize Hg concentrations. The values represented in the boxplots are the first quartile, median, and third quartile. The whiskers are the minimum and maximum values and the diamonds represent the arithmetic mean.

Exploratory analyses were performed where log-transformed total Hg concentrations in adobe brick, dirt floor, surface dust, and vapor samples were compared between neighborhoods using one-way ANOVA. Tukey’s Studentized Range (HSD) test was used to identify all pairwise comparisons that were statistically significantly different controlling for the experiment wise error rate. In addition, Pearson correlation coefficients, denoted by r, were calculated to estimate the dependence of sample types on a neighborhood level and Huancavelica-wide. No adjustment was made for multiple comparisons in testing the correlations between sample types. All statistical analyses were completed using SAS 9.2 software (SAS Institute Inc., Cary, NC).

## Results

### Total Hg in adobe bricks, dirt floors, and surface dust

**Figure 2:**
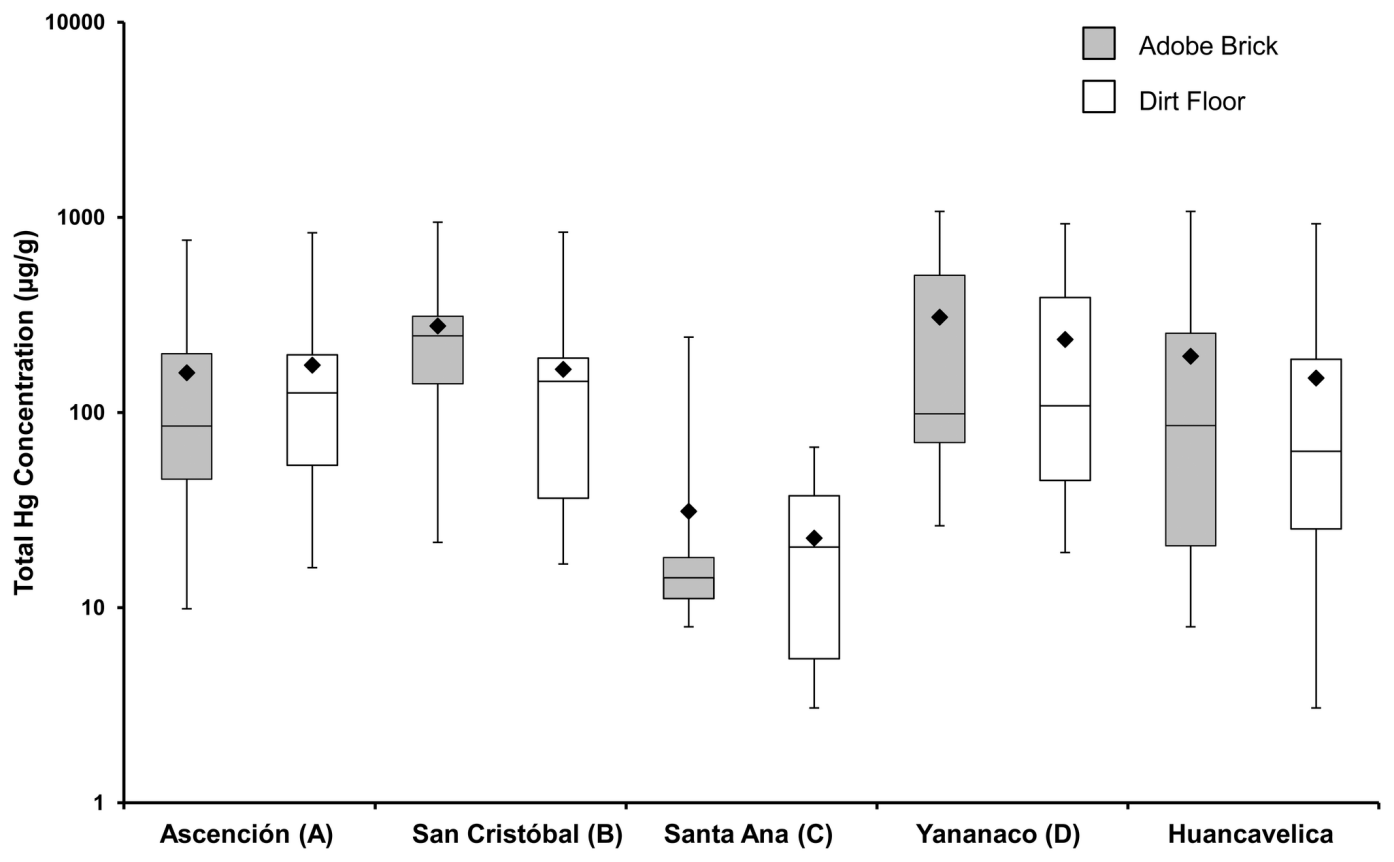
Total Hg concentrations in adobe bricks and dirt floors by neighborhood.

**Figure 3:**
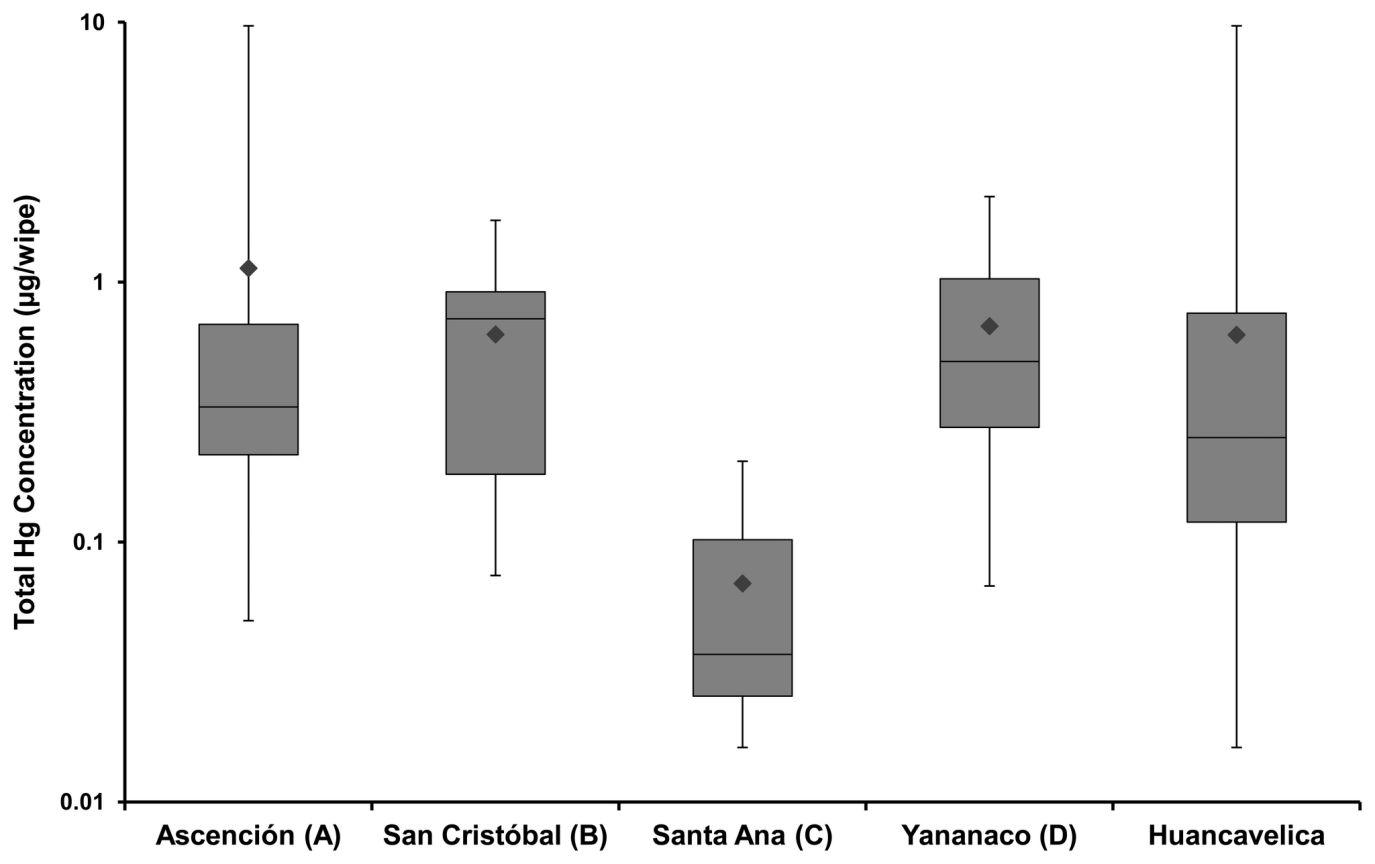
Total Hg concentrations in surface dust by neighborhood.

The total Hg concentrations in the adobe bricks and dirt floors from 15 homes in each of the four neighborhoods are shown in Figure 2. The figure shows that total Hg concentrations in the adobe bricks and dirt floors from the 60 households ranged from 8.00 to 1070 µg/g and 3.06 to 926 µg/g, respectively. The median (10^th^ and 90^th^ percentile) total Hg concentrations in adobe bricks and dirt floors were 85.5 µg/g (11.3 µg/g and 563 µg/g, respectively) and 63.3 µg/g (6.99 µg/g and 369 µg/g, respectively). In comparison, the total Hg concentrations in exterior adobe brick and ambient soil samples from the reference site, Ayacucho, ranged from 0.03 to 0.57 µg/g and 0.07 to 0.15 µg/g, respectively. The total Hg concentrations in surface dust wipes across the 60 households are shown in Figure 3 and ranged from 0.02 to 9.69 µg/wipe. The median (10^th^ and 90^th^ percentile) total Hg concentrations in surface dust were 2.25 µg/g (0.03 µg/g and 1.09 µg/g, respectively).

### Elemental Hg vapor in indoor air

**Figure 4:**
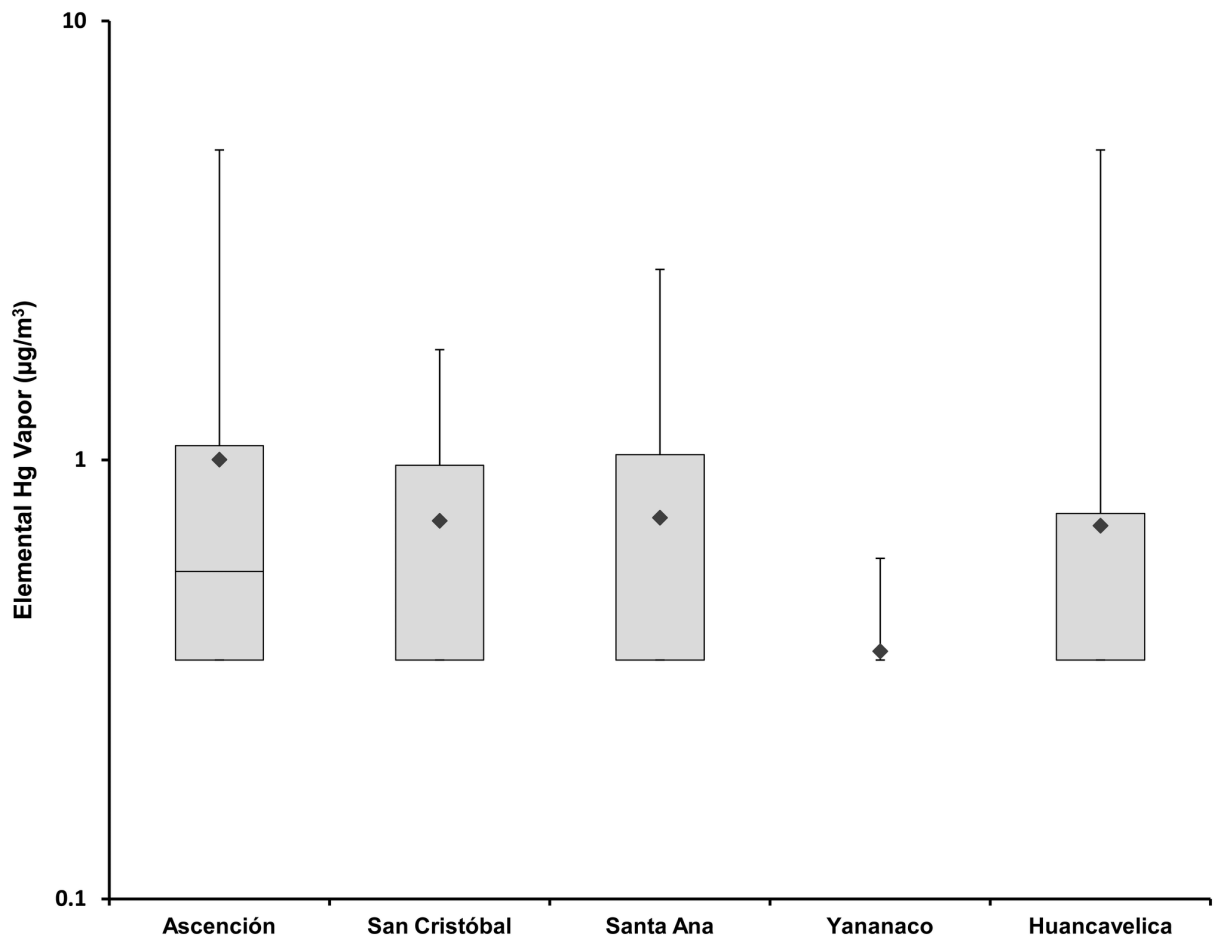
Elemental Hg vapor concentrations in indoor air by neighborhood.

Figure 4 shows the elemental Hg vapor concentrations that were measured in 15 homes from each of the four neighborhoods, using the Jerome J405 Mercury Vapor Analyzer. Thirty nine of the 60 households were below the limit of detection of the instrument. Elemental Hg vapor concentrations across the 60 households ranged from 0.35 to 5.1 µg/m^3^.

## Discussion

### Differences in total Hg in adobe bricks, dirt floors, and surface dust between neighborhoods

Results of the one-way ANOVA for adobe bricks, dirt floors, and surface dust suggest that a significant difference exists in the total Hg concentrations according to the neighborhood (p<0.0001). Results of the Tukey’s HSD test for adobe brick samples, dirt floor samples and for surface dust samples indicate that Ascensión (A), San Cristóbal (B), and Yananaco (D) were not significantly different from one another; however, Santa Ana (C) had significantly lower Hg concentrations in adobe bricks, dirt floors, and surface dust than the other three neighborhoods (p<0.0001).

As shown in Figure 1, the neighborhoods of Ascensión (A), San Cristóbal (B), and Yananaco (D) historically had smelters operating throughout the neighborhood [4,6]. Santa Ana (C), in contrast, did not have any smelters during the colonial refining period. The lower concentrations of total Hg in adobe bricks, dirt floors, and surface dust in Santa Ana may be due to the absence of historic cinnabar refining in this section of the city.

### Relationships between total Hg in adobe bricks, dirt floors, and surface dust

Pearson correlation coefficients were calculated for total Hg concentrations in adobe bricks, dirt floors, and surface dust on a neighborhood and Huancavelica-wide basis, as shown in Table 1. A strong correlation was observed in Yananaco (D) between total Hg concentrations in adobe bricks and dirt floors (r=0.766, p<0.001). Moderate correlation was observed between these sample types in Ascención (A) (r=0.545, p=0.036) and San Cristóbal (B) (r=0.500, p=0.058). These results suggest a significant correlation between total Hg concentrations in adobe bricks and dirt floors in Ascensión (A) and Yananaco (D). Moderate correlation was observed between total Hg concentrations in surface dust and dirt floors in San Cristóbal (B) (r=0.539, p=0.038) and Yananaco (D) (r=0.593, p=0.020). A statistically significant correlation was not found between total Hg concentrations in surface dust and adobe bricks in any of the individual neighborhoods.

**Table 1:** Pearson correlation coefficients, sample size, and p-value for log-transformed total Hg concentrations in adobe bricks, dirt floors, and surface dust by neighborhood.

Neighborhood	Sample Size	Sample Type	Adobe Brick	Dirt Floor	Surface Dust
Ascención (A)	15	Adobe Brick	1.00		
		Dirt Floor	0.545 (p=0.036)*	1.00	
		Surface Dust	0.214 (p=0.443)	0.477 (p=0.072)	1.00
San Cristóbal (B)	15	Adobe Brick	1.00		
		Dirt Floor	0.500 (p=0.058)	1.00	
		Surface Dust	0.510 (p=0.052)	0.539 (p=0.038)*	1.00
Santa Ana (C)	15	Adobe Brick	1.00		
		Dirt Floor	0.402 (p=0.138)	1.00	
		Surface Dust	-0.006 (p=0.982)	0.122 (p=0.666)	1.00
Yananaco (D)	15	Adobe Brick	1.00		
		Dirt Floor	0.766 (p<0.001)*	1.00	
		Surface Dust	0.461 (p=0.084)	0.593 (p=0.020)*	1.00
Huancavelica	60	Adobe Brick	1.00		
		Dirt Floor	0.720 (p<0.001)*	1.00	
		Surface Dust	0.589 (p<0.001)*	0.685 (p<0.001)*	1.00

* indicates statistical significance at α=0.05; p-values not corrected for multiple comparisons

Table 1 shows that the correlations between all sample types become statistically significant (p<0.001) when Pearson correlation coefficients are calculated for total Hg concentrations in adobe bricks, dirt floors, and surface dust across all 60 residences in Huancavelica. The strong correlation between total Hg concentrations in adobe bricks and dirt floors (r=0.720, p<0.001) confirms what the residents of Huancavelica reported: that they build adobe bricks using the materials present on their property rather than purchase manufactured adobe bricks made from materials external to their property. Moreover, the moderate correlation coefficients for total Hg concentrations in surface dust and adobe bricks (r=0.589, p<0.001) and surface dust and dirt floors (r=0.685, p<0.001) across the entire community suggest that adobe bricks and dirt floors are the primary source of Hg in surface dust inside the residences. Because many homes in Huancavelica are built from on-site adobe, these results indicate the potential for widespread Hg contamination throughout several neighborhoods.

Pearson correlation coefficients were also calculated city-wide after excluding Santa Ana (not shown in Table 1) because total Hg concentrations in adobe brick, dirt floors, and surface dust were found to be significantly lower in Santa Ana than in the other four neighborhoods. When Santa Ana is excluded, moderate correlation was observed for the remaining households (n=45) for adobe bricks and dirt floors (r=0.591, p<0.001), surface dust and adobe bricks (r=0.349, p<0.001), and surface dust and dirt floors (r=0.529, p<0.001). Although the correlation coefficients are lower when excluding Santa Ana, the correlations between sample types are still very significant.

Previous studies of Hg concentrations in residential surface dust have primarily focused on the contribution of outdoor sources of Hg in more developed parts of the world. Lemus et al. [10] measured Hg concentrations in household dust and outdoor soil in urban and rural homes in south Louisiana. Concentrations of Hg in household dust ranged up to 157 µg/g and 175 µg/g for urban and rural houses, respectively. For outdoor soil, concentrations of Hg ranged up to 125 µg/g and 82.2 µg/g for urban and rural houses, respectively. The authors estimated an indoor to outdoor mean ratio for Hg of 0.44 based on arithmetic means, which suggested that Hg in household dust was coming from an outside source.

In areas close to historical Hg mining locations such as Idrija, Slovenia, elevated Hg levels in dust have also been observed [11]. In that study, the median Hg concentrations in attic dust and soils near homes in Idrija were 129 µg/g and 47 µg/g, respectively, for the closest and most contaminated area (Area 1). The authors found that Hg concentrations decreased with distance from the source of contamination, with median Hg concentrations in attic dust (Area 2: 17 µg/g, Area 3: 6.1 µg/g) and soils (Area 2: 3.2 µg/g, Area 3: 1.0 µg/g) well below Area 1. The authors found a strong relationship between Hg concentrations in attic dust and soils, suggesting outdoor sources of Hg influenced indoor dust concentrations.

Likewise, in a study in Ottawa, Canada, Rasmussen et al. [12] measured Hg in indoor dust and exterior soils and dust in Ottawa, Canada. Average Hg concentrations in garden soil and street dust were 0.107 µg/g and 0.029 µg/g, respectively, while average house dust Hg concentrations were 3.633 µg/g. The authors found that indoor sources contributed to Hg concentrations in house dust more than outdoor sources.

Rasmussen et al. [12] also suggested that, because of higher concentrations of biogenic particles and organic matter in indoor dust compared to outdoor soil and dust, there is potential for indoor dust to accumulate higher concentrations of Hg and other metals. The authors suggest that this accumulation, a result of poor air exchange particularly in damp homes, can lead to an increase in fungi in indoor dust, which can accumulate greater concentrations of Hg. This finding is particularly important in Huancavelica, where homes are often damp and poorly ventilated. Because the adobe brick walls and dirt floors in Huancavelica are typically unsealed and subjected to dampness and inadequate air exchange, there is potential for walls and floors, in addition to surface dust, to accumulate greater concentrations of Hg than a home with sealants applied to walls and floors.

### Differences in elemental Hg vapor between neighborhoods

Results of the one-way ANOVA for elemental Hg vapor suggest that there is not a significant difference in elemental Hg vapor concentrations based on neighborhood (p=0.126). The Tukey’s HSD test for elemental Hg vapor confirms that there are not significant differences between neighborhoods at a significance level of α=0.05. Hg vapor is relatively mobile, so may move more readily than dust or dirt. This finding may also be a reflection of the large number of measurements that were below the limit of detection of the instrument (39 of 60 homes) and suggests that, while some individual households appear to have elevated elemental Hg vapor concentrations, these variations cannot be attributed to differences in characteristics of the four neighborhoods.

### Implications for potential health effects from exposure

The semi-arid climate of Huancavelica results in a very dusty ambient environment stemming from many unpaved roads and outdoor surfaces. Results of a previous study have identified ambient total Hg concentrations in surface soil in Huancavelica ranging between 1.75 and 698 µg/g [4]. Results of the present study found similar or greater concentrations of total Hg in adobe bricks, dirt floors, and surface dust. While health effects resulting from Hg exposure depend on the exposure pathway and the type of Hg present, it is clear that the residents of Huancavelica can be exposed to Hg through both outdoor and indoor environments. Robins et al. [4] suggest that, in ambient soil, a majority of the Hg present is a sulfur-bound mineral phase (e.g., cinnabar or metacinnabar). While other forms of Hg, such as organic and elemental Hg, have more immediate and severe health implications, all species of Hg pose a potential health risk to those exposed.

In the dusty, widely contaminated community of Huancavelica, it is important to recognize that ingestion of particle-bound Hg may affect health. A previous study demonstrated that children are especially vulnerable due to their hand-to-mouth activity [13]. An 11 kg, 1 year old toddler with a median ingestion rate of 100 mg of dirt and dust from hand-to-mouth activity per day [14] would exceed EPA’s Reference Dose (RfD) for inorganic mercury (0.3 mg kg^-1^ day^-1^) [15] if the soil contained more than 33 µg Hg/g soil. For the 60 households studied in Huancavelica, 40 adobe brick, 42 dirt floor, and 30 surface dust samples had total Hg concentrations above 33 µg/g. The dirt and dust ingestion rate used in the EPA study were representative of children in the United States. It is likely that children in Huancavelica, and the Andean region in general, are smaller than children in the United States and, because adobe walls and dirt floors are typically unsealed and the community is generally dusty, children in Huancavelica could potentially ingest more dirt and dust from hand-to-mouth activity than the average child in the United States. If so, then the calculations above associated with ingesting dirt and dust for children in Huancavelica may underestimate the potential risks.

This study demonstrates that building materials used widely in the developing world, such as adobe bricks, may be a substantial source of residential Hg exposure in contaminated communities. In Huancavelica, Hg in adobe brick homes is a result of legacy contamination that primarily occurred hundreds of years ago during the Spanish colonial period. Understanding exposure to Hg from such building materials, particularly adobe bricks made in the community, has broader relevance to evaluating risks in communities that currently produce or refine silver and gold, such as Madre de Dios and Puerto Maldonado, both also in Peru. In these communities, gold shops and other buildings used for refining are often constructed from adobe brick that has the potential to act as a sink for elemental Hg vapor released from current refining processes and may be a source of Hg exposure in the future.

## Conclusions

The purpose of this study was to determine the current levels of mercury to which residents of adobe brick homes in Huancavelica are exposed. Results from this study suggest that total Hg concentrations in residential samples are similar to or greater than ambient soil samples from previous studies conducted in Huancavelica, and that widespread residential exposure to Hg is presently occurring there. Total Hg concentrations ranged from 8.00 to 1070 µg/g in adobe bricks, 3.06 to 926 µg/g in dirt floors, and 0.02 to 9.69 µg/wipe in surface dust. These results indicate that total Hg concentrations vary by neighborhood, with Santa Ana (C) having consistently lower Hg concentrations than the other three neighborhoods. This finding may suggest that the present-day spatial distribution of total Hg contamination in residences is influenced by the location of historical cinnabar smelters.

These results also point to a strong relationship between total Hg concentrations in adobe bricks and dirt floors. This result confirms that residents of Huancavelica are using materials on their property to construct adobe bricks for their homes, rather than purchasing manufactured adobe bricks. Additionally, the strong relationship between total Hg concentrations in surface dust and adobe bricks and surface dust and dirt floors suggests that adobe bricks and dirt floors are the primary source of Hg in indoor dust, rather than an outdoor source.

Elemental Hg vapor concentrations from 39 of the 60 residences in Huancavelica were below the limit of detection. For residences with measurements above the limit of detection, concentrations of elemental Hg vapor ranged up to 5.1 µg/m^3^. While individual homes vary in Hg vapor concentrations, no significant difference in these concentrations was found between neighborhoods.

The results of this study demonstrate that adobe bricks and other building materials used in developing communities may be a substantial source of residential Hg exposure, particularly in communities with past or present mercury production and where silver or gold refining utilizes Hg for amalgamation. Adobe bricks are used to construct buildings around the world, from the southwest United States to South America to Africa. As the first study of adobe brick contamination anywhere in the world, the present study draws attention to a previously unidentified source of potential exposure to Hg and possibly to other contaminants. To better understand potential health effects from exposure, it is important to quantify levels of contaminants, identify the chemical species and bioaccessibility of contaminants in the building materials, and to identify possible mitigation strategies to reduce exposure within these homes.
